# Claimed Effects, Outcome Variables and Methods of Measurement for Health Claims on Foods Related to Vision Proposed Under Regulation (EC) 1924/2006

**DOI:** 10.3390/nu10020211

**Published:** 2018-02-14

**Authors:** Daniela Martini, Augusto Innocenti, Chiara Cosentino, Giorgio Bedogni, Donato Angelino, Beatrice Biasini, Ivana Zavaroni, Marco Ventura, Daniela Galli, Prisco Mirandola, Marco Vitale, Alessandra Dei Cas, Riccardo C. Bonadonna, Giovanni Passeri, Carlo Pruneti, Daniele Del Rio

**Affiliations:** 1 The Laboratory of Phytochemicals in Physiology, Department of Food and Drug, University of Parma, 43125 Parma, Italy; daniela.martini@unipr.it (D.M.); donato.angelino@unipr.it (D.A.); beatrice.biasini@studenti.unipr.it (B.B.); 2 Department of Medicine and Surgery, Clinical Psychology Unit, University of Parma, 43125 Parma, Italy; augusto.innocenti@unipr.it (A.I.); chiara.cosentino1@unipr.it (C.C.); 3 Clinical Epidemiology Unit, Liver Research Center, Basovizza, 34149 Trieste, Italy; giorgiobedogni@gmail.com; 4 Department of Medicine and Surgery, Division of Endocrinology, University of Parma, 43125 Parma, Italy; ivana.zavaroni@unipr.it (I.Z.); alessandra.deicas@unipr.it (A.D.C.); riccardo.bonadonna@unipr.it (R.C.B.); 5 The Azienda Ospedaliera Universitaria of Parma, 43125 Parma, Italy; 6 Laboratory of Probiogenomics, Department of Chemistry, Life Sciences and Environmental Sustainability, University of Parma, 43125 Parma, Italy; marco.ventura@unipr.it; 7 Department of Medicine and Surgery, Sport and Exercise Medicine Centre (SEM), University of Parma, 43125 Parma, Italy; daniela.galli@unipr.it (D.G.). prisco.mirandola@unipr.it (P.M.); marco.vitale@unipr.it (M.V.); 8 Department of Medicine and Surgery, University of Parma, Building Clinica Medica Generale, 43125 Parma, Italy; giovanni.passeri@unipr.it

**Keywords:** health claim, outcome variable, method of measurement, diet, vision

## Abstract

Adequate visual function has a strong impact on the quality of life of people. Several foods and food components have been hypothesized to play a role in the maintenance of normal visual function and in the prevention of eye diseases. Some of these foods/food components have been the object of a request of authorization for use of health claims under Articles 13(5) or 14 of the Regulation (EC) 1924/2006. Most of these requests have received a negative opinion from the European Food Safety Authority (EFSA) due to the choice of inappropriate outcome variables (OVs) and/or methods of measurement (MMs) applied in the studies used to substantiate the claims. This manuscript refers to the collection, collation and critical analysis of OVs and MMs related to vision. Guidance document and requests for authorization of health claims were used to collect OVs and MMs related to vision. A literature review was performed to critically analyse OVs and MMs, with the aim of defining their appropriateness in the context of a specific claimed effect related to vision. The results highlight the importance of adequate choices of OVs and MMs for an effective substantiation of claims related to visual function.

## 1. Introduction

Proper vision development and setup are features with strong impacts on practically all aspects of an individual’s life. Blindness and low vision, are recognized as important causes of impairment in high income countries [1]. A recent review has shown that, in high income countries, as well as in in Eastern and Central Europe, blindness and mild and severe vision impairment (MSVI) decreased from 1990 to 2010 from 0.2 to 0.1% (3.314 million to 2.736 million people) and from 1.6 to 1.0% (25.362 million to 22.176 million), respectively [2]. However, MSVI can still lead to dramatic consequences, particularly for younger people. Vision loss, is linked to (i) depression in people more than 20 years old; (ii) a substantial economic burden, particularly for the those who are less than 40 years old [3,4] and (iii) to a dramatic change in the way people perceive their own ability to function independently, hence, in their quality of life [5].

Uncorrected refractive error, followed by cataracts, macular degeneration, glaucoma and diabetic retinopathy, are the most common causes of MSVI. Beyond diabetes, which is one of the biggest nutritional contributors to complete vision loss, a wide range of ocular defects have their origin either in nutritional deficiencies or excess. These conditions have been shown to respond favourably to nutritional components [6].

In this scenario, some food components have been the object of applications for authorization of health claims relevant to Regulation (EC) 1924/2006. Most of these applications have received a negative opinion from the European Food Safety Authority (EFSA), for several reasons that fall into one of these three categories: (i) insufficient characterization of the food/food component; (ii) choice of an inappropriate claimed effect and (iii) insufficient substantiation of the claim, including inadequate sample size or statistical analysis, or choice of inappropriate outcome variables (OVs) and/or methods of measurement (MMs). Based on this consideration, a project has been developed to collect, collate and critically analyse OVs and MMs that have been proposed for the substantiation of health claims, with the aim of improving the quality of applications submitted by applicants to EFSA. Previous papers related to different areas of interest have been already published [7,8,9,10,11]. This manuscript refers to the collection, collation and critical analysis of OVs and MMs related to vision.

## 2. Materials and Methods: Search Strategy

Guidance document (EFSA 2012), requests for authorization of health claims under Articles 13.5 and 14 of Regulation 1924/2006 related to visual function and comments received during public consultations were screened to collect OVs and MMs. As already described [7], OVs and related MMs were considered only if the food/food constituent(s) were sufficiently characterized and if the claimed effect was considered to be well defined and beneficial. Following this decision tree, one claimed effect with six OVs was evaluated under Article 13.5, whereas two claimed effects with a total of five OVs referred to children development were selected under Article 14.

For the collation, a specific syntax with appropriate keywords was used to create a database of references on PubMed for each OV. These databases were used to critically analyse OVs and MMs which were ranked as: (i) appropriate; (ii) appropriate only in combination with other OVs or MMs; (iii) not appropriate per se; (iv) not appropriate in relation to the specific claimed effect proposed by the applicant(s); (v) useful as supportive evidence for the substantiation of the claimed effect.

Regarding the MMs, all the methods proposed for each OV in the scientific opinion and/or in the guidance document were included in the evaluation, unless the related OV was considered to be inappropriate per se, or for the specific claimed effect. If no methods were proposed or all the proposed methods were considered inappropriate, the best or the most widely used methods were added.

## 3. Results: Critical Analysis of Outcome Variables and Methods of Measurement Proposed for Health Claims Related to Visual Function

### 3.1. Function Health Claims Article 13 (5)

#### 3.1.1. Maintenance or Reduced Loss of Visual Function

##### 3.1.1.1. Visual Acuity

Visual acuity (VA) commonly refers to clarity of vision and is the measure of the spatial resolution of the visual processing system. It mainly depends on how light is focused on the retina, the integrity of the retina and the interpretation of the cognitive brain, and it declines with age. VA is affected by the form and dimensions of stimuli, the number of them, the distance between the stimuli and the subject, the lightness, the contrast with the background, etc.

In the second half of the 19th Century, Snellen was the first to design special characters (“optotypes”) for assessing VA. Normal vision was defined by Snellen as “the ability to recognize an optotype from a distance of 20 feet when it subtends an angle of 1 arc min”. Snellen designed his optotypes as letters-with-serifs on a 5 × 5 grid. About a century later, Sloan designed optotypes as non-serif letters on a 5 × 5 grid. The later British standard also recommends non-serif letters, but on a 4 × 5 grid.

To evaluate the appropriateness of VA as the OV of maintenance or reduced loss of visual function, the literature derived from database #1 was critically evaluated (Table 1).

VA is the most commonly used measure of visual function and it is applied in both clinical evaluations and research studies. Many scales have been used to measure VA in different countries, so that different definitions of normal vision exist [12]. This lack of standardization has been the major problem in comparing VA data for more than a century. About 40 years ago, the International Council of Ophthalmology proposed a standardization in the testing of the VA by adopting the Early Treatment of Diabetic Retinopathy Study (ETDRS) chart.

As mentioned above, “normal” visual acuity is often defined by considering the definition proposed by Snellen. Based on Snellen’s chart, normal acuity is designated as 6/6 m, 20/20 feet (in the United States), 1.00 decimal or 0.0 log Minute of Arc (MAR). It has been shown that the average visual acuity of an emmetropic eye (or ametropic eye with correction) in young humans is approximately 6/5 to 6/4. Therefore, referring to 6/6 visual acuity as the “perfect” vision may be inaccurate. The visual acuity needed to discriminate two contours separated by 1 arc min (equivalent to 1.75 mm at 6 m) is 6/6. In other words, the 6/6 standard can be considered the lowest limit of normal VA, so that individuals reaching this level need no further investigation. However, it is worth noting that normal VA does not necessarily reflect normal visual function in individuals with other visual problems, such as amblyopia.

Measurement of VA can be performed by using an eye chart, optical instruments, or computerized tests [13]. To obtain reliable results, it is important to consider the conditions corresponding to the standard, including the correct illumination of the room and the eye chart, as well as the correct viewing distance. European countries have standardized these conditions through the norm, EN ISO 8596.

In conclusion, VA alone is an appropriate and universally accepted outcome variable to assess visual function and thus it can be used for the substantiation of claims in the context of maintenance or reduced loss of visual function.

3.1.1.1.1. Early Treatment Diabetic Retinopathy Study

ETDRS uses the Bailey and Lovie layout with five Sloan optotypes for line and a standard test distance of 4 m, back illuminated to a calibrated light level, and requires a detailed protocol for counting each correctly identified letter. Optotypes need to be calibrated against the Landolt ‘C’ that represents the reference optotype, as indicated by the International Council of Ophthalmology in 1984 [14].

The main characteristic of the charts proposed by Bailey and Lovie is the proportional layout given by the letter spacing equal to the letter/symbol width and to the line spacing equal to the height of the lower line, combined with a logarithmic progression. Due to these two characteristics, these charts are sometimes called “logMAR” charts, even though logarithmic progression was already proposed by Green in the previous century.

Although the ETDRS-type layout often uses Roman letters, non-Roman letters or other symbols can also be used.

The main advantages of the Green/Bailey-Lovie/ETDRS charts are the constant step size at all levels of vision and the constant crowding and contour interaction given by the proportional layout with equal numbers of letters on each row [13].

The charts were designed for a 4-m distance between the chart and the subject. Therefore, they can be easily used at 1–2 m. An ETDRS chart reduced by 10 times in size can be used in Near Visual Acuity (NVA) assessment (at 40 cm distance), providing an exact correlation with VA at 4 m in subjects with normal vision.

The ETDRS test incorporates specific design criteria, including an equal number of letters per row, and an equal spacing of letters and rows on a log scale. This makes ETDRS more accurate than the Snellen or Sloan acuity tests. Moreover, different versions of the ETDRS test chart are available to reduce problems related to memorization. The three standard versions of the ETDRS chart are R, 1 and 2.

The chart should be presented with standard illumination and both frontlit and backlit versions are available. Moderate variations in illumination are allowed. However, despite the estimation that doubling the illumination will make only a one-letter difference, findings of scientific studies show that changes in illuminance can significantly impact VA, thus contributing to test–retest variability [15].

Because of differences in the task of recognizing single letters and a letter embedded in a chart, the letters should not be pointed at or presented in isolation.

ETDRS chart and the associated protocol are considered by the International Council of Ophthalmology as the gold standard for visual acuity testing and they are used as a research standard, so they can be considered the best method for the measurement of VA and NVA.

##### 3.1.1.2. Near Visual Acuity

NVA is the measure of visual acuity at a “reading distance” and is typically evaluated at a standard distance of 40 cm. Despite the principles of geometrical optics, in some clinical and subclinical conditions visual acuity at a distance and in proximity are not the same (i.e., presbyopia) [16].

To evaluate the appropriateness of NVA as an OV of maintenance or reduced loss of visual function, the literature deriving from database #1 was critically evaluated (Table 1).

The measurement of NVA has the same rules as that of VA (See VA, Section 3.1.1.1). To compare distance and near visual acuity tests, test conditions, optotypes and chart design must be the same. The standard test distance is set at 40 cm and provides an easy correlation with a 4-m testing distance. The use of different distances is allowed even if the actual test distance should be measured and specified in all instances.

A reduction of the dimension of chart used in the distance VA by 10 times provides an exact correlation between NVA (40 cm) and VA (4 m).

As well as for VA, the International Council of Ophthalmology proposed, in 1984, a standardization of the evaluation of NVA adopting the ETDRS chart.

In conclusion, NVA alone is an appropriate and universally accepted outcome variable to assess visual function and, thus, it can be used for the substantiation of claims in the context of maintenance or reduced loss of visual function.

##### 3.1.1.3. Contrast Sensitivity

Contrast is a measure of the amount of light or darkness an object has against its background. The smallest difference in light intensity or darkness between an object and its background that can be distinguished is defined as contrast threshold. Contrast sensitivity (CS) is the inverse of contrast threshold and, therefore, differs from VA which is a measure of the spatial resolving ability of the visual system under conditions of very high contrast [17]. CS is generally expressed in log units to make the values linear and allow comparisons at low and high levels of contrast. The amount of contrast can be quantified using the Weber or Michelson formulas. The Weber formula is used when the background luminance remains constant. When both light and dark components change, the Michelson formula should be used.

To evaluate the appropriateness of CS as an OV of maintenance or reduced loss of visual function, the literature derived from database #1 was critically evaluated (Table 1).

The ability to detect objects at a low level of contrast is closely related to the skill of performing tasks such as driving and reading and, hence, it must be considered a primary aspect in the evaluation of visual performance. CS provides additional information about the quality of vision. CS is mainly measured by applying chart-based systems using test targets that are either sine-wave gratings or letters (optotypes).

In conclusion, CS is an appropriate and universally accepted outcome variable for assessing visual function, thus, it can be used for the substantiation of health claims in the context of maintenance or reduced loss of visual function.

3.1.1.3.1. Pelli–Robson Contrast Sensitivity Chart

The Pelli–Robson Chart is the most used letter chart for assessing CS. It is a wall-mounted chart, made of 16 triplets of Sloan letters, arranged in eight rows with six letters (i.e., two sets of triplets) per row. Each letter subtends a visual angle of 2.86 at a 1-m test distance. The contrast between the test letters progressively decreases from ~100% at the top to less than 1% at the bottom of the chart, in 0.15-log unit sensitivity steps for each triplet of letters. The chart is printed on both sides so separate charts with different letters are used for testing each eye. The subject’s task is to read the letters aloud starting at the top and moving through the chart until he or she cannot read anymore. A score ranging from 0 to 2.25 (corresponding to log CS) is given based on the reading capacity of the subject. In the original scoring system, 0.15 credits per triplet are provided when at least two of the three letters are read correctly. However, modified scoring systems also exist [18].

The Pelli–Robson Chart has a high test–retest reliability and the scores are relatively unaffected by background luminance, testing distance (from 0.25 to 4 m), and until 2 dioptres of defocus.

Thanks to the brevity and ease of administration (3–5 min) and the availability of published normative data, letter charts, such as the Pelli–Robson Chart, show good test–retest reliability. These advantages often lead to a preference for these tests for letter contrast sensitivity over sine-wave gratings tests [19].

In conclusion, the Pelli–Robson Chart is the gold standard method for measuring CS in healthy individuals.

##### 3.1.1.4. Sensitivity/Disability Glare

Glare is a visual sensation caused by excessive and uncontrolled brightness, such as direct or reflected sunlight, or artificial light (e.g., car headlamps at night), which makes vision difficult. Two types of glare can be distinguished: discomfort and disability glare. Discomfort glare can be distracting but it is not necessarily associated with impaired vision. Conversely, disability glare causes impaired vision even if it is not necessarily associated with discomfort. As defined by the International Commission on Illumination (CIE), disability glare is “the visual conditions in which there is excessive contrast or an inappropriate distribution of light sources that disturbs the observer or limits the ability to distinguish details and objects”. In general, the effect of glare increases with an increasing glare source or with a decrease in background luminance. Disability glare seems to be particularly important in mesopic vision, which is a combination of photopic vision (e.g., under high light levels, typical during the day) and scotopic vision (i.e., under very low light levels), in dim lighting situations. Mesopic vision, in combination with glare, seems to be stable until age 40 and then gradually decreases, whereas mesopic vision without glare starts to decrease at around 51–60 years. Therefore, glare leads to an earlier decrease in mesopic vision, as compared to non-glare conditions [20].

To evaluate the appropriateness of sensitivity/disability glare as an OV of maintenance or reduced loss of visual function, literature derived from database #1 was critically evaluated (Table 1).

Disability glare is the result of intraocular light scattering due to a nearby glare source and leads to a loss of retinal image contrast, and so, to a reduced VA [21]. Disability glare could be explained by neural, as well as by physical factors. From the neural point of view, the photoreceptors need a period of light adaptation, followed by a reduction in contrast in the retinal image, because of the intraocular light scatter. On the other hand, the normal light scattering properties of the eye play a role in the aetiology of disability glare. Actually, disability glare occurs in cases of projection of a glare source onto a blind area of the retina, supporting the stray light hypothesis [20].

On the basis of current literature, sensitivity/disability glare does not appear to be an appropriate outcome variable for assessing visual function so it cannot be used alone for the substantiation of claims on maintenance or reduced loss of visual function. However, it can be useful as supportive evidence in the assessment of visual function.

3.1.1.4.1. Straylight Meter

As already mentioned, disability glare represents the effect of retinal contrast reduction due to the veil of the retinal straylight. In this context, CIE defined disability glare as the straylight. Retinal straylight can be measured by the straylight meter, an instrument that relies on a dedicated psychophysical technique for straylight assessment. Originally, it was based on the direct compensation method, in which the amount of straylight is measured by using a variable counterphase compensation light that is present in the test field.

More recently, straylight measurement has been based on the comparison compensation method, with the central test field including two half fields, one with, and one without, counterphase compensation light [22].

In addition to the straylight meter, other methods have been developed to measure disability glare. In these tesst, the effect of glare on VA or CS is taken to represent the subject’s “glare sensitivity”. Over the years, many glare testers have been proposed for assessing visual functions like VA or CS in the presence of a glare source. However, none of these have been classed as the standard method for evaluating disability glare, so the straylight meter is still considered the gold standard.

The straylight meter differs from the other glare methods because it directly measures the relevant physical magnitude affecting the eye. Conversely, the sensitivity value determined by other glare testers may be affected by other ocular factors, including the state of dark adaptation [23]. In conclusion, the straylight meter can be considered the gold standard method for measuring glare sensitivity/disability.

##### 3.1.1.5. Visual Field

Visual field is the term used to identify the area one sees while the eye is fixed to one point. It represents the area within which information can be perceived without moving the head and eyes. The normal vision field consists of an island of central vision, which includes the inner 30 degrees, and the peripheral visual field, that extends 100 degrees laterally (temporal), 60 degrees medially (nasal), 60 degrees upward, and 70 degrees downward. The visual field could be split in two vertical areas: nasal and temporal hemi-fields. In the temporal hemi-field, about 12 to 17 degrees from fixation and 1.5 degrees below the horizontal meridian, is situated the normal blind spot. The blind spot anatomically corresponds to the scleral canal through which the retinal nerve leaves the eye, while the binocular visual field is composed of the overlapping of the two monocular fields. [24].

To evaluate the appropriateness of visual field as an OV of maintenance or reduced loss of visual function, the literature derived from database #1 was critically evaluated (Table 1).

Among the main diseases affecting vision, visual field loss and visual acuity loss are probably the most severe. Visual field loss may occur independently from visual acuity loss. Visual field loss could be due to many clinical conditions (i.e., glaucoma, retinopathies, strokes etc.), but numerous studies have reported an increased incidence in the elderly. A linear decline in the visual field seems to start around the age of 20 until ages 50 to 60, with a slightly greater reduction in sensitivity afterward. Consequences of visual field loss are very important for orientation and mobility performance [25]. Most studies on the visual field in healthy populations have been conducted to evaluate its correlation with driving performance. A variety of tests and evaluation procedures are available, but the widely used assessment of visual field is perimetry (either kinetic or static).

In conclusion, measurement of the visual field is an appropriate outcome variable for assessing visual function and therefore it can be used for the substantiation of health claims in the context of maintenance or reduced loss of visual function.

3.1.1.5.1. Perimetry

Perimetry is a useful quantitative method for visual field evaluation, by means of either kinetic or static assessment. Overall, various techniques and devices are available, all including a confrontation field testing. While individuals maintain steady visual fixation straight ahead, objects with different sizes, colours, or luminosities transcend their visual threshold. Although the first perimetric tests were conducted manually, automated perimetry systems have now been developed. In fact, the drawback of manual perimetry falls in the subjectivity of manual presentation of the stimulus by the operator and recording of the patient’s response to the stimulus. In contrast, automated perimetry allows a standard presentation of the stimulus and standard recording of responses, leading to more reproducible results. Manual perimetry is still useful for individuals who cannot adapt well to the automated interface.

In static perimetry, the threshold of vision at a particular point within the visual field is detected by means of stimuli with different intensities. The combination of threshold sensitivities defines the subject’s visual field. An automated version of the static perimetry test (e.g., Humphrey visual field analyser), which provides more standardized testing conditions, is the most widely used visual field examination. However, this testing algorithm shows high variability and a ‘‘learning curve effect” [26].

Kinetic perimetry (e.g., Goldmann kinetic manual perimetry) is one of the oldest techniques used for visual field testing, and it is characterized by an operator who determines the area of the visual field by the use of a moving stimulus with a fixed intensity [27]. Points of equal sensitivity are connected to form an isopter, representing the outer limit for that stimulus intensity. The test is repeated for various stimulus intensities, and obtained isopters are combined to identify the areas of central and peripheral visual fields. Kinetic perimetry is the method of choice in cases of both advanced and early stages of visual field loss. Kinetic testing is more sensitive than conventional automated static perimetry for detecting and monitoring peripheral visual field defects. Moreover, it has a greater flexibility for dynamic evaluation of the visual field and its results correlate better with activities of daily living. Kinetic perimetry is preferred over static perimetry in individuals with poor compliance, in children, and for detecting small, multiple defects in the visual field periphery. A semi-automated kinetic perimetry (e.g., Octopus 900 perimeter) has been developed, allowing computer-controlled standardized presentation for any combination of Goldmann stimulus, in any direction over the whole visual field. Furthermore, the standardization of kinetic perimetry results is to be increased, thanks to the used of predefined starting and ending points for the stimulus vectors and constant angular velocities.

Both Humphrey and Octopus are recommended by the European Glaucoma Society for routine diagnosis of glaucoma.

In conclusion, perimetry testing is an appropriate method to be used for the measurement of the visual field.

##### 3.1.1.6. Photostress Recovery

Photostress recovery is a measure of the time that the macula requires to return to its normal level of function after being exposed to a bright light source (photostress). The reduction in visual acuity results from photopigment depletion, whereas the recovery time depends on the rate of photopigment regeneration [28].

To evaluate the appropriateness of photostress recovery as OV of maintenance or reduced loss of visual function, the literature derived from database #1 was critically evaluated (Table 1).

Photostress recovery allows the differentiation between normal and abnormal macular function. Photostress recovery is not specific for a certain disease, but there are different clinical conditions that can affect the functioning of the macular area [29]. For instance, in some cases, macular integrity can be related to visual function, but this relationship is not univocal. In other words, photostress recovery is a measure of the macular integrity and not a measure of visual function.

In conclusion, photostress recovery is not an appropriate outcome variable for assessing visual function and, thus, it is not appropriate for the substantiation of health claims concerning the maintenance or reduced loss of visual function.

### 3.2. Claims Referring to Children’s Development and Health Art 14(b)

#### 3.2.1. Maintenance and Support of Eye Development

##### 3.2.1.1. Visual Evoked Potential Acuity

Visual Evoked Potential Acuity (VEP) is the electrical potentials that are generated in the visual cortex when the retina is stimulated with light [30]. The electroencephalogram signal is typically used to extract VEP waveforms, by using the ensemble averaging technique. VEP is mainly applied to measure the visual pathway integrity from the retina to the visual cortex. Moreover, it allows VA to be accessed in the case of infants or lack of cooperation from the patient. The stimuli used to generate VEPs can be both unpatterned or patterned (i.e., checkerboard or grating). The responses elicited by the former show lower intra- and inter-individual variability, so they are generally preferred, with a few exceptions (i.e., in cases of uncooperative patients) [31]. Among the pattern stimuli, checkerboard pattern reversal is the most widely used because of its relative simplicity and reliability. VEP acuity refers to acuity of vision measurement by VEP techniques. VEPs have been used to assess the vision of infants and young children since the 1970s [32].

To evaluate the appropriateness of VEP acuity as an OV of maintenance and support eye development, the literature derived from database #2 was critically evaluated (Table 1).

In the early years of life, the maturation of the visual process is accompanied by continuous morphological changes in the retina, as well as in the optic nerve and visual cortex, and VEP is often used to assess the development of normal visual function. Studies suggest a deep difference between visual function in children compared to adults. These differences are mainly ascribable to the deficient spatial vision in infants and to the immaturity of the fovea, the retinal region that supports high resolution. VEPs, as psychophysiological measures of infants’ VA (i.e., Tellers Acuity Carts), assess grating acuity, a visual task based on the resolution ability of the visual system. VEP acuity is therefore a widely used outcome variable in the assessment of development of the visual system and its functions [33].

In conclusion, VEP acuity is an appropriate outcome variable for measuring eye development and visual development in infants and young children and can be used for the substantiation of related health claims.

3.2.1.1.1. Sweep Visual Evoked Potential

Sweep Visual Evoked Potential (sVEP) is a relatively recent technique to assess VA in infants and non-verbal children. It was first proposed by Norcia, in 1985 [33], and it is essentially a steady state VEP, on which a pattern of elements that varies in some aspect over time. sVEP evaluates VA and CS over a shorter recording time compared to the conventional pattern. Because no cooperation is needed, sVEP is particularly useful for visual acuity testing in infants, non-verbal children, individuals with a short attention span, as well as for studying the development of vision [34]. To this aim, a stimuli pattern that is alternated at a high temporal frequency rate, producing a steady state visual evoked response, is generated by the program. Real-time measurement of the amplitude and phase of the response is then possible, thanks to a discrete Fourier transform that is performed on the recorded signals. To allow for VA measurement, the size of the pattern is quickly reduced. The spatial frequency is swept by presenting many different patterns within few seconds. This allows the estimation of VA from the smallest pattern size that produces a response.

In conclusion, sVEP is the best technique for measuring VEP acuity in infants and children, for whom it is the gold standard.

##### 3.2.1.2. Stereo Acuity

Binocular vision is a condition in which the two eyes are involved in the formation of a single image, allowing the subject to integrate monocular images to build a single image with higher quality. The monocular retinal images are slightly different, because the eyes are distant from them and therefore have a slightly different orientation with respect to the observed object. This discrepancy is known as retinal disparity. An efficient binocular vision requires the retinal image of the two eyes to be in focus, and both images must be of similar shape and dimension. Moreover, the eyes should be able to coordinate with each other so that the retinal image can easily be maintained in the fovea by both eyes simultaneously. For each point of fixation, the visual system calculates the retinal disparity between the objects making up the visual space and assigns them a greater or lesser depth. Thus, retinal disparity, produced by the distance between the two eyes, is the basis of depth perception. The visual-perceptive process that leads to the sensation of depth, starting from the two monocular retinoic images is named stereopsis, and stereo acuity is the smallest detectable disparity that can be discriminated. Stereopsis is achieved at a cortical level, where the information from the two eyes are reworked. Stereoscopic capability starts to become refined a few weeks after birth, due to postural reflexes and the development of fusion reflex. It is completed at around six months of age, when it can be considered equal to that of adults. It develops earlier in females than in males [35].

To evaluate the appropriateness of stereo acuity as an OV of maintenance and support eye development, the literature derived from database #2 was critically evaluated (Table 1).

Disorders of vision, like amblyopia and strabismus, as well as ocular diseases which reduce visual acuity, may damage stereo acuity. Stereo acuity has been proposed as a reference for assessing good visual health. Stereo acuity is mainly used in clinical assessment, but, because of its sensitivity to the development of stereoscopic capability, it can be considered a good secondary outcome in research on the development of the visual system.

In conclusion, the stereo acuity “per se” is not an appropriate outcome variable in measuring eye development in infants and young children. However, it can be used as supportive evidence of other visual outcomes (e.g., VA, VEP acuity etc.).

3.2.1.2.1. Random Dot Test

Julesz first introduced the Random Dot Test in 1960 [36]. There are several types of Random Dot Stereo Tests. In Random Dot Tests, stereo acuity is measured from the subject’s ability to identify forms from random dot backgrounds (stereogram) presented on several cards or pages of a book. Patient should recognize a figure present in a stereogram.

Random Dot Tests have shown good validity for detecting amblyopia, strabismus and suppression, and in stereo acuity assessment [37].

Particularly, it can determine subtle changes in stereo acuity and it has a good sensitivity to loss of stereo acuity [38].

In conclusion, the Random Dot Test is an appropriate method for measuring Stereo acuity in infant and children.

#### 3.2.2. Support Visual Development

##### 3.2.2.1. Visual Evoked Potential Acuity

See Section 3.2.1.1.

3.2.2.1.1. Sweep Visual Evoked Potential

See Section 3.2.1.1.1.

3.2.2.1.2. Pattern Reversal Visual Evoked Potential

Pattern reversal VEP is the standard clinical test for evaluating the integrity of visual pathways. The test is based on a black-and-white checkboard crossing the central 20–30° of the visual field. The VEP is based on the response of the subject to the periodical change of black/white square positions. The responses are then recorded using three electrodes spanning the occipital region, with a mid-frontal electrode as the voltage reference.

Drawbacks of the method mainly consist in the need for complete cooperation from the volunteer, as the checkboard has to be fixed and focused. Moreover, it cannot be used in newborn infants and in children or adults who cannot focus or who cannot understand or follow instructions.

The recording procedure is longer and complex than sVEP; thus, in infants and young children, the assessment of VEP acuity using sVEP is preferable [39].

In conclusion, pattern reversal VEP is an appropriate technique for measuring VEP acuity in infants and children, but it cannot be used in newborn infants and non-cooperative children.

3.2.2.1.3. Flash Visual Evoked Potential

Flash VEP is a way to record VEP following the exposure of a flash generated by a stroboscope, which will cover the entire visual field, not considering the light source point. This technique was utilized to record VEP in newborn infants and in children and adults that cannot focus or are not cooperative.

The main drawback of such recordings is the high variability of the VEP flash, which does not allow the retrieval of precise and specific information about the signal received by the brain [40].

This concern mainly depends on the additional pathways reaching the retinoic-striate projection, as VEPs are generated in response to a flash, not only by the striate area, but also by cortical regions.

Regarding newborns, variability is much higher because of the maturation of the cerebral cortex. For this reason, this method does not provide reliable information on the retinoic–striate conduction time, lacking specificity for addressing the quality or prognosis of visual perception [41].

In conclusion, flash VEP is not an appropriate technique for measuring VEP acuity in infants and children, due to its high variability.

##### 3.2.2.2. Visual Acuity

See Section 3.1.1.1.

3.2.2.2.1. Teller Acuity Cards

Teller Acuity Cards represent a tool used for assessing visual acuity in newborns and infants. They are based on the “preferential looking” technique, which investigates the reaction of subjects undergoing different visual stimuli. If the child gazes, turns the head towards the stimulus, or moves the eyes, then a response to these stimuli is occurring. By observing a child’s reaction to a certain stimulus, it is possible to assess his/her visual–perceptive threshold. The Teller Acuity Card procedure allows rapid evaluation of grating VA in different populations, such as infants, young children and subjects who cannot communicate.

When the visual acuity of infants and young children is standardly assessed in laboratory settings, different acuity cards are shown to the subject on a grey screen, which has the role of minimizing distractions in infants and young children during VA assessment. The screen presents attached side panels, while a grey shield is suspended at a distance of 38 or 55 cm from the screen [42].

Compared to other techniques, such as optokinetic nystagmus and VEP, as well as to other “preferential looking” tests, the Teller Acuity Cards should be preferred because of their high reliability. Moreover, these cards have the advantage of being used in subjects of different ages, from birth until the end of the preschool period. Furthermore, they are quick and simple to use.

Teller Acuity Cards has been validated in many studies and showed good long-term reliability and predictive validity [43].

In conclusion, Teller Acuity Cards are the best method for measuring VA in infant and children, and they are the gold standard for infants and young children.

##### 3.2.2.3. Retinal Development

In newborns, the eye has a dioptre apparatus, suitable for the proper refraction of light rays and the proper focus of images on the retina, but the retina and the fovea are not fully developed. Two different types of photoreceptor cells (i.e., rods and cones) can be found in the retina. The functional organization of the retina provides a scotopic system consisting of rods and a photopic constituted by cones. The scotopic system is deputy to the vision in low light conditions. The photopic system is delegated to daylight vision and colour perception. The fovea is a hollow circle in the retina, with the greatest concentration of the red and green cones, while rods are completely absent. The fovea is the part of the retina where VA is a higher. At birth, VA and CS are much lower than in adults. They grow very fast in the first few months of life, but after 6 months, the rate of development slows down [44].

To evaluate the appropriateness of retinal development as an OV of support visual development, the literature derived from database #2 was critically evaluated (Table 1).

In newborns, the fovea is not differentiated, and begins to develop in the first few months of life. Retinal development implies the formation and growth of foveal cones, a maturation of their external segments and an increase of their synapses.

The development of the retina, however, is by itself insufficient to explain the increase in VA and visual functions that occur in the first year of life. In fact, even the changes that occur in the central nervous system contribute to this. These changes involve the lateral geniculate body, visual cortex and their synaptic connections. Therefore, visual development is related to the maturation of the retina, particularly the fovea, and the rise in receptive fields of neurons along the visual pathways [45].

In conclusion, retinal development is an appropriate outcome variable in measuring visual development in infants and young children and, thus, can be used for the substantiation of health claims on visual development.

3.2.2.3.1. Electro-Retino-Gram

The electro-retino-gram (ERG) represents the electrical response of the retina induced by a light stimulus. It is represented by a complex waveform that results from the interaction of the electric potentials generated by the different retinal components. The response of the scotopic and photopic systems are evaluated separately by changing the state of adaptation of the retina and using different light stimuli [46].

ERG is widely used clinically to assess retinal function because may show an abnormal response in retinal disorders, even when there are no detectable abnormalities on funduscopic examination. It has also been shown that ERG reflects retinal development. In fact, changes in the ERG response during an infant’s growth reflect different developmental stages in photoreceptors, middle retinal layers and more proximal retina [47].

In the uncooperative infant or child, it is important to be able to record ERG without the need for sedation or anaesthesia.

In conclusion, ERG is an appropriate technique for measuring retinal development in infants and children.

## 4. Conclusions

Insufficient scientific substantiation of a health claim is one of the most common reasons for a negative response to applications for authorization of a health claim.

This work critically analyses all the OVs and MMs that have been proposed so far in the context of maintenance of vision relevant to Regulation (EC) 1924/2006, as summarized in Table 2. However, independently from the critical analysis of each OV and MM, the use of a battery of OVs (for instance visual acuity and contrast sensitivity) is always preferable, to obtain clearer and more exhaustive evidence on the effect of a food/food component on visual function.

The information provided by this work could be used by stakeholders during the design of randomized controlled trials aimed to substantiate health claims related to vision. Moreover, EFSA could use the information to update the related guidance on the scientific requirements for health claims.

However, it is worth repeating that randomized controlled trials should take into account many parameters other than appropriate OVs and related MMs, including an adequate sample size, a proper study design and an adequate statistical analysis.

## Figures and Tables

**Table 1:** Strategies used for retrieving the literature pertinent with outcome variables and methods of measurement related to cognitive function in adults.

DB Number	Syntax	Total Articles	Narrative Reviews	Systematic Reviews/Meta-Analyses	Validation Studies	Outcome Variables
1	“vision, ocular”[mesh] OR “visual perception”[mesh] OR “vision tests”[mesh]) AND “English”[language] AND “humans”[mesh]	167,577	7554	1239	637	Visual acuity Near visual acuity Contrast sensitivity Glare sensitivity/disability Visual field Photostress recovery
2	(“evoked potentials, visual”[mesh] OR “electroretinography”[mesh] OR “vision development”[title/abstract] OR “visual acuity”[mesh] OR “visual development”[title/abstract] OR “eye development”[title/abstract]) AND “English”[language] AND “humans”[mesh]	69,219	2450	696	265	Visual evoked potential acuity Stereo acuity Retinal development

**Table 2:** Summary of the main findings of the study. Legend: A: appropriate itself; S: only supportive; N: not appropriate for that claimed effect; /: not evaluated (because the outcome variable is not appropriate per se).

CLAIMED EFFECT	OUTCOME VARIABLE(S)	CRITICAL ANALYSIS	METHOD(S)	CRITICAL ANALYSIS
3.1. FUNCTION HEALTH CLAIMS ART 13 (5)				
*3.1.1. MAINTENANCE OR REDUCED LOSS OF VISUAL FUNCTION*	Section 3.1.1.1. Visual Acuity	A	3.1.1.1.1. Early Treatment Diabetic Retinopathy Study	A
	3.1.1.2. Near Visual Acuity	A	3.1.1.2.1. Early Treatment of Diabetic Retinopathy Study	A
	3.1.1.3. Contrast Sensitivity	A	3.1.1.3.1. Pelli–Robson Contrast Sensitivity Chart	A
	3.1.1.4. Glare Sensitivity/Disability	S	3.1.1.4.1. Straylight Meter	A
	3.1.1.5. Visual Field	A	3.1.1.5.1. Perimetry	A
	3.1.1.6. Photostress Recovery	N	/	/
3.2. CLAIMS REFERRING TO CHILDREN’S DEVELOPMENT AND HEALTH ART 14(b)				
*3.2.1. MAINTENANCE AND SUPPORT OF EYE DEVELOPMENT*	3.2.1.1. Visual Evoked Potential Acuity	A	3.2.1.1.1. Sweep Visual Evoked Potential	A
	3.2.1.2. Stereo Acuity	A	3.2.1.2.1. Random Dot Test	N
*3.2.2. SUPPORT VISUAL DEVELOPMENT*	3.2.2.1. Visual Evoked Potential Acuity	A	3.2.2.1.1. Sweep Visual Evoked Potential	A
			3.2.2.1.2. Pattern Reversal Visual Evoked Potential	A
			3.2.2.1.3. Flash Visual Evoked Potential	N
	3.2.2.2. Visual Acuity	A	3.2.2.2.1. Teller Acuity Cards	A
	3.2.2.3. Retinal Development	A	3.2.2.3.1. Electro-Retino-Gram	A
